# Letter to the editor: New metrics to monitor progress towards global HIV targets: using the estimated number of undiagnosed HIV-infected individuals as denominator

**DOI:** 10.2807/1560-7917.ES.2016.21.50.30424

**Published:** 2016-12-15

**Authors:** André Sasse

**Affiliations:** 1 Scientific Institute of Public Health, Brussels, Belgium

**Keywords:** HIV infection, PLHIV, undiagnosed, modelling, diagnosis, YDF

**To the** **editor**: Recently, Pharris et al. presented an important analysis of the current HIV situation in the European Union/European Economic Area (EU/EEA), focussing on the estimates produced by the European Centre for Disease Prevention and Control (ECDC) HIV Modelling Tool, and especially the number of people living with HIV who are not aware of their infection [1]. 

The ECDC HIV Modelling tool allows estimation of the number of undiagnosed HIV-infected individuals by country, by transmission route, or at European level [2,3]. This number is commonly compared with the total number of people living with HIV (PLHIV); the ratio of both numbers forms the undiagnosed fraction, providing information on the first of the Joint United Nations Programme on HIV/AIDS (UNAIDS) 90–90–90 global targets for 2020 ‘90% of persons living with HIV diagnosed’ [4]. However, in countries where a large proportion of patients are in care, this ratio may be an insufficient indicator of testing and diagnosis success because PLHIV on treatment currently live longer with HIV and consequently, the proportion of undiagnosed persons with HIV will naturally become smaller in relation to the ever-increasing population of diagnosed PLHIV [1]. In a population with a large number of successfully treated patients, even a very high diagnosed fraction of 90% may hide a large and increasing number of undiagnosed infections that may sustain HIV transmission in the near future. Hence, the diagnosed fraction does not provide precise enough information to gauge testing services’ effectiveness.

However, the estimated number of undiagnosed PLHIV can contribute to a more effective indicator of diagnosis timeliness and completeness. Used as a denominator, the number of undiagnosed PLHIV could be an appropriate indicator of quality of testing programmes when it is compared with the yearly number of new diagnoses. This comparison may be expressed as the ratio of yearly number of new diagnoses to the estimated number of undiagnosed individuals, or as a fraction, the yearly diagnosed fraction (YDF), expressed as follows: yearly number of new diagnoses / (yearly number of new diagnoses + estimated number of undiagnosed PLHIV). To monitor testing programmes, comparing the yearly number of diagnoses with the number of undiagnosed individuals is likely to provide more sensitive and dynamic information than the diagnosed fraction calculated among the total population of PLHIV.

Looking at today’s epidemic in the EU/EEA, what would be the current value of the YDF?

Considering the HIV epidemic in EU/EEA, the estimated number of PLHIV who are not aware that they are infected is 122,000, or 15% of all PLHIV [1]. In 2015, 29,747 people were newly diagnosed with HIV [5]. The yearly number of new HIV diagnoses remains quite stable over time. The calculated YDF is 20% (29,747 / (29,747 + 122,000)). It means that only one in five of the estimated number of PLHIV susceptible to be diagnosed in 2015 was actually diagnosed in 2015. At constant number of diagnoses, four years at least would be necessary to diagnose the number of still undiagnosed PLHIV (not taking into account those expected to become infected during coming years), leading to many late diagnoses, and consequently fuelling HIV transmission. In fact, the diagnoses of the people who are currently unaware of their infection will likely take place over a longer period of time.

In order to reduce HIV transmission and improve individual and population health, the undiagnosed PLHIV should be diagnosed and offered treatment as soon as possible [6], ideally becoming the early diagnosed of tomorrow rather than the late diagnoses of after-tomorrow. Increases in the number of diagnoses would be something positive if there was a concomitant increase in YDF. A considerable increase in the number of diagnoses, and therefore in YDF, would be necessary to cut the cycle of infection-transmission. So an ideal situation would be that all persons currently living with undiagnosed HIV receive a diagnosis next year (YDF = 100%).

The YDF is an indicator that relates ‘work done’ (persons diagnosed this year) to ‘work remaining to be done’ (persons undiagnosed). The value of YDF and its trend over time are indicators that can monitor the situation and progress on HIV testing and timely HIV diagnosis, thus helping guide efforts to reducing the number of undiagnosed PLHIV and curbing the epidemic.
